# A Hidden Catastrophe: Ruptured Abdominal Aortic Aneurysm Presenting as Acute Ischemic Stroke

**DOI:** 10.7759/cureus.116531

**Published:** 2026-09-19

**Authors:** Eric Malani, Lucy Maguire, Maxwell Blodgett

**Affiliations:** 1 Emergency Medicine, Christiana Care Health System, Newark, USA; 2 Neurocritical Care, Thomas Jefferson University, Philadelphia, USA

**Keywords:** abdominal aortic aneurysm, embolic stroke, ischemic stroke, stroke treatment, ultrasonography

## Abstract

A ruptured abdominal aortic aneurysm (AAA) is a life-threatening emergency that typically presents with hypotension or abdominal pain but rarely with primary neurological deficits.

We report the case of a 74-year-old woman presenting with acute focal neurological deficits concerning for ischemic stroke, whose preceding abdominal pain and lower extremity mottling ultimately pointed toward an alternative diagnosis. Point-of-care ultrasound and subsequent computed tomography angiography revealed a ruptured infrarenal AAA. We discuss how consideration of these additional clinical features led to the correct diagnosis and prevented inappropriate thrombolytic therapy. Given the time-sensitive nature of stroke management, this case highlights the importance of maintaining a broad differential diagnosis and considering aortic pathology in patients presenting with acute neurological symptoms.

## Introduction

Abdominal aortic aneurysm (AAA) is a relatively common vascular condition defined by progressive dilation of the abdominal aorta due to degenerative weakening of the aortic wall. Greater than three centimeters is a commonly accepted threshold for diagnosis of an AAA. Risk factors including advanced age, smoking, male sex, and atherosclerosis contribute to degradation of the vascular media and extracellular matrix, promoting aneurysmal expansion and increasing the risk of rupture. If these aneurysms are identified early, typical initial treatment focuses on risk factor modifications. If an aneurysm reaches more than 5.5 cm in diameter, or is expanding at a rate of more than 0.5 cm per six months, operative repair is often considered [1,2]. Despite its potentially catastrophic consequences, AAA is frequently asymptomatic and is often discovered incidentally on imaging performed for unrelated indications [3]. When symptoms do occur, they are typically related to aneurysm expansion or rupture. Patients with enlarging AAAs may report abdominal, flank, or back pain, while rupture can present with hypotension and abdominal pain, although many patients with ruptured AAAs do not survive to reach medical care [1,4].

While the association between aortic dissection and cerebrovascular events is well described [5-7], the relationship between AAA and cerebrovascular events is less clearly established, and stroke as a presenting manifestation of AAA is rarely reported. Two prior case reports of similar atypical AAA presentations were identified [8,9]. In both, the ruptured AAA was recognized hours into the emergency department (ED) stay only after additional clinical deterioration. In one case, the patient had already received tenecteplase (TNK) at the time of ruptured AAA diagnosis and died within hours [8,9].

We present a case of AAA identified in the setting of acute neurological deficits concerning for ischemic stroke, highlighting an overall rare and underrecognized clinical presentation.

## Case presentation

A 74-year-old female with a history of tobacco use presented to the ED at 10:50 AM via emergency medical services (EMS), minimally responsive and with mottling of the bilateral lower extremities. She was in her normal state of health that morning prior to the acute onset of left lower quadrant abdominal pain, vomiting, and collapsing in the bathroom, at which point EMS was activated. Upon EMS arrival, she was noted to have altered mental status and lower extremity mottling. Her last known well time was estimated at 10:15 AM.

On arrival at the ED, the patient had a blood pressure of 140/103 mmHg with a pulse of 105. She was noted to be minimally responsive, with a leftward gaze deviation, right-sided hemiparesis, complete aphasia, and inability to follow commands. She demonstrated withdrawal of the left upper and lower extremities to painful stimuli but was unable to cooperate with commands to assess extremity drift. Her Glasgow Coma Scale was 7 (Eyes 2, Verbal 1, Motor 4). Her skin was diffusely mottled in the lower extremities. Her cardiopulmonary examination was unremarkable, and her abdomen was described as soft and nondistended. A stroke alert was activated; her initial NIHSS was 24, and she was intubated for airway protection. Given the reported preceding abdominal pain and mottled extremities on examination, a point-of-care aortic ultrasound was obtained, demonstrating a dilated abdominal aorta when measured from outer wall to outer wall in the transverse plane (Figure 1). The patient was taken for CT head and CT angiography (CTA) of the head, neck, chest, abdomen, and pelvis. CT head without contrast was unremarkable. CT stroke protocol imaging was notable for 50% stenosis of the left common carotid artery origin, a supraclinoid left internal carotid artery aneurysm measuring 3 mm, and no large vessel occlusion. CTA of the chest, abdomen, and pelvis demonstrated an acute rupture of the abdominal aorta, with hemorrhagic fluid seen in the retroperitoneum on both sides of the aorta. The aorta was dilated up to 5.3 × 5.0 cm over a distance of at least 15 cm, from around the level of the renal arteries to the level of the bifurcation (Figure 2). Laboratory data were significant for a creatinine level of 1.54 mg/dL, with an unclear baseline, and mild leukocytosis/anemia, with WBC of 14.8 × 10³/μL and HGB of 10.5 g/dL. Platelet count, PT, and INR were unremarkable at 358 × 10³/μL, 12.8 seconds, and 1.1, respectively. The balance of the complete blood count and basic metabolic panel was also unremarkable.

**Figure 1:**
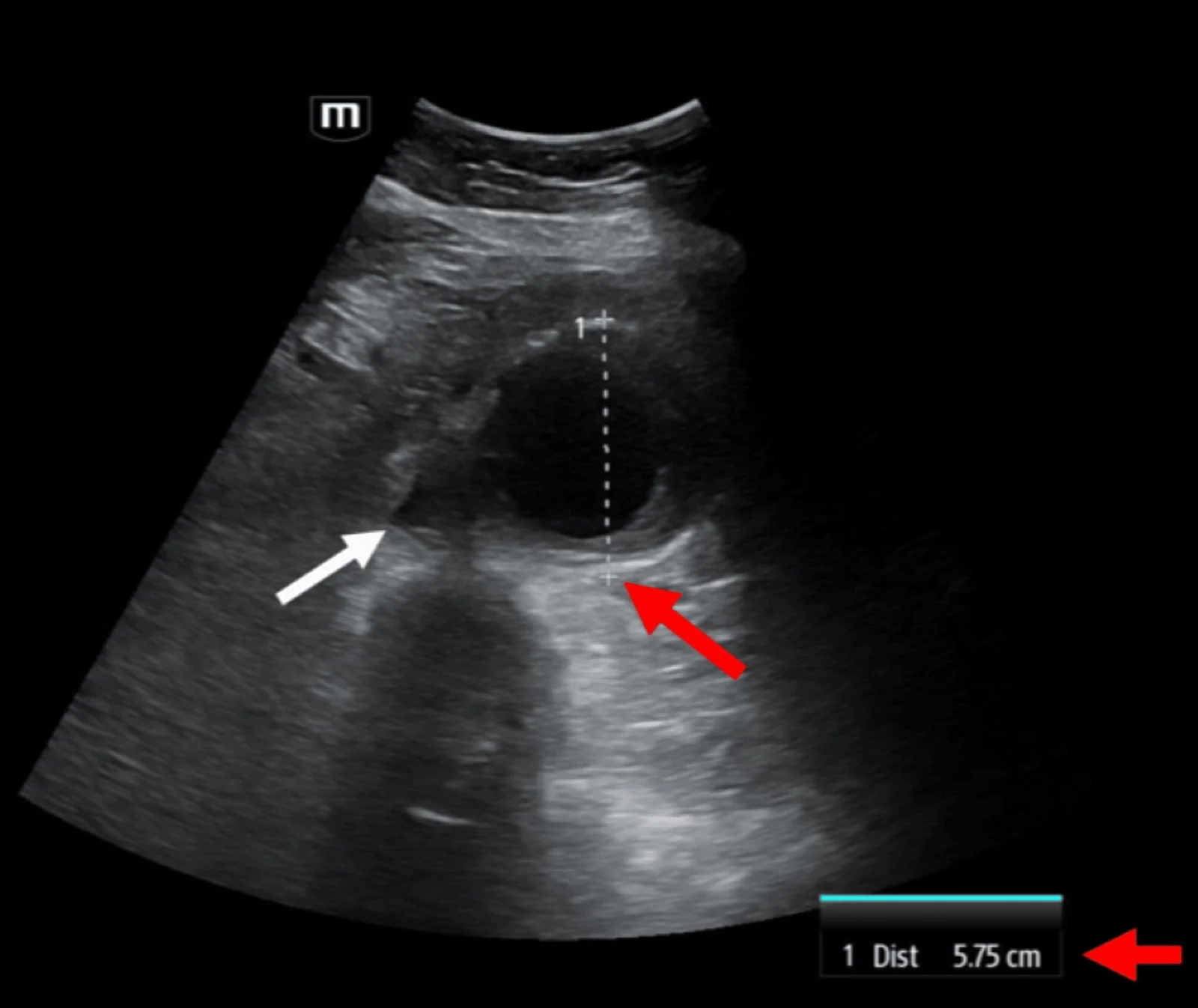
Point of Care Ultrasound (POCUS) Transverse Aorta Bedside ultrasound demonstrated a dilated abdominal aorta measuring 5.75 cm in anterior-posterior diameter (red arrows). Ultrasound imaging also demonstrated a hypoechoic para-aortic area, indicating an abdominal aortic aneurysm suggestive of possible rupture, which was later confirmed on computed tomography angiography (CTA) (white arrow).

**Figure 2:**
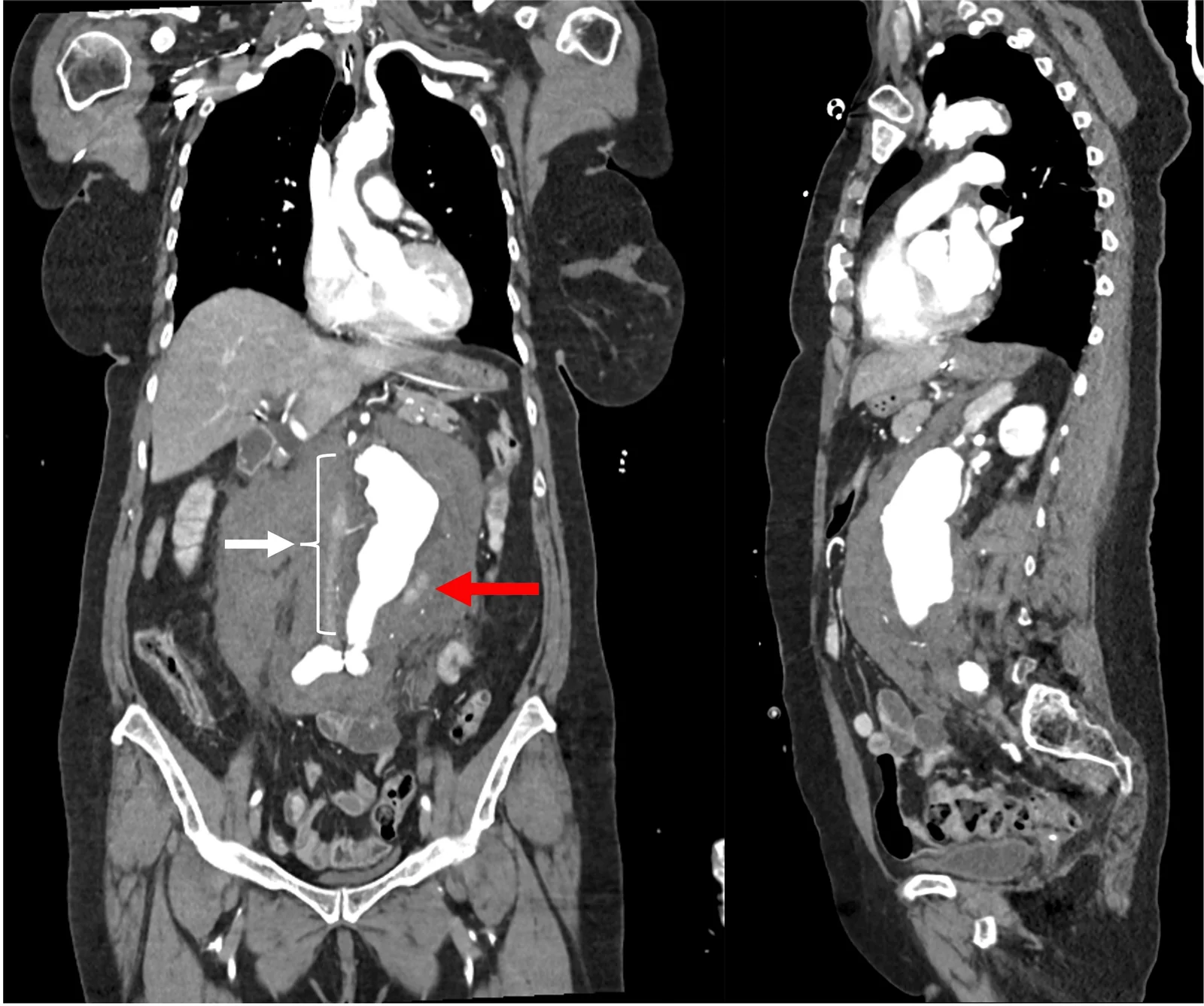
CTA Aorta Coronal and Sagittal Computed tomography angiography (CTA) with contrast of the abdominal aorta obtained during initial stroke alert imaging. CTA imaging demonstrated a ruptured infrarenal abdominal aorta with adjacent hemorrhage and hematoma (red arrow) as well as aneurysmal dilation of the infrarenal abdominal aorta from the renal arteries to the bifurcation (white arrow and bracket).

Although the patient was within the TNK administration window, TNK was not administered due to AAA rupture suspected on bedside ultrasound (Figure 1) and subsequently confirmed on CT imaging (Figure 2). CT images were reviewed in real time, and a STAT consult was placed with vascular surgery. The patient was then taken emergently for endovascular aneurysm repair (EVAR) using a Medtronic Endurant II bifurcated stent graft system for a confirmed ruptured infrarenal AAA with a contained retroperitoneal hematoma. Hemodynamic stability was maintained throughout the procedure, estimated blood loss for the case was 150 mL, and the patient was subsequently admitted to the surgical ICU for continued monitoring. Postoperative laboratory tests demonstrated a worsening WBC count to 26.9 × 10³/μL, HGB to 8.2 g/dL, and creatinine to 1.68 mg/dL. Lactic acid was also found to be elevated to 5.1 mmol/L at this time. The patient’s systolic blood pressure ranged from 92 to 155 mmHg on admission, which was mostly consistent with her systolic goal of 100-130 mmHg. She was treated with 81 mg of aspirin daily immediately after EVAR and clopidogrel 75 mg daily for five days postoperatively. The timing of dual antiplatelet therapy initiation for stroke was decided based on collaborative recommendations from vascular surgery and neurology. The patient had a follow-up MRI two days after initial presentation, which demonstrated multiple foci of acute infarct in the left frontal, parietal, and occipital lobes, as well as small foci of acute infarct in the right frontal lobe and the left cerebellum (Figure 3). While the infarcts were labeled as acute, it is unclear if they occurred preoperatively or postoperatively, since MRI was obtained after completion of EVAR. Over the next several days, she had continued improvement in aphasia and right-sided weakness and was ultimately discharged to a skilled nursing facility on hospital day 10. The patient’s neurologic examination at the time of discharge demonstrated partial gaze palsy with no effort against gravity in the right arm, some effort against gravity in the left arm and bilateral legs, and no aphasia or dysarthria. NIHSS at discharge was 11, and the Modified Rankin Scale was 4, as she continued to require assistance with walking.

**Figure 3:**
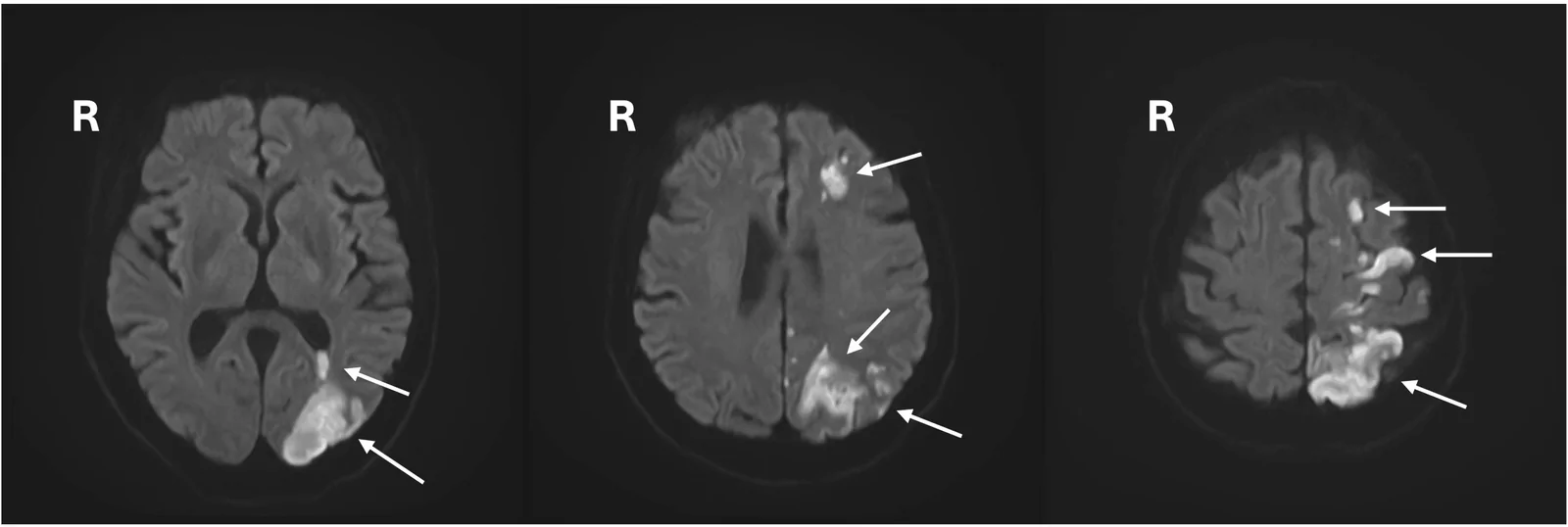
MRI Axial DWI Magnetic resonance imaging (MRI) with diffusion-weighted imaging (DWI) obtained on hospital day 2 demonstrated multiple foci of acute infarction in the left frontal, parietal, and occipital lobes, with additional small foci of acute infarction scattered in the right frontal lobe and left cerebellum (white arrows). Images from left to right demonstrate ascending MRI slices from caudal to cranial.

## Discussion

This case highlights the diagnostic and management challenges that arise when acute neurological deficits occur in the setting of life-threatening aortic pathology. Although ischemic stroke is a common neurologic emergency, the differential diagnosis for acute focal deficits must remain broad, particularly when historical features or physical findings suggest alternative etiologies. Our patient presented with classic signs of a severe stroke: gaze deviation, aphasia, and hemiparesis. However, the preceding history of sudden abdominal pain and the presence of bilateral lower extremity mottling raised concern for a concurrent vascular catastrophe. Bedside ultrasound revealed a widened abdominal aorta, and subsequent computed tomography angiography demonstrated a ruptured infrarenal AAA. This case illustrates the importance of maintaining a high index of suspicion for aortic pathology when evaluating patients with acute neurologic deficits, as there are competing therapeutic priorities between ischemic stroke (thrombolysis) and ruptured AAA.

Neurological manifestations are well described in association with aortic dissection, where stroke may occur when the dissection flap propagates into the carotid or vertebral arteries and compromises cerebral perfusion. Neurologic symptoms such as focal deficits, syncope, and altered mental status have been reported in up to one-third of patients with dissection [10]. In contrast, stroke is an uncommon initial presentation of AAA [8,9]. The mechanism linking AAA to cerebrovascular events remains incompletely understood. In this case, the presence of infarcts in multiple vascular territories on MRI suggests an embolic mechanism. However, echocardiography demonstrated normal ejection fraction and left atrial size, with no patent foramen ovale identified; EKG showed normal sinus rhythm, and no arrhythmia was identified on telemetry while inpatient. While other case reports of AAA and stroke have also suggested an embolic source based on MRI [8], further research is necessary to elucidate the pathophysiology. 

This case also highlights the diagnostic complexity of thrombolytic decision-making in the hyperacute stroke setting. Intravenous thrombolytics such as alteplase and TNK are effective therapies for acute ischemic stroke when administered within the recommended treatment window. However, these medications carry a significant risk of hemorrhage. In patients with undiagnosed vascular catastrophes such as ruptured AAA, administration of thrombolytics could lead to fatal bleeding. Our patient initially appeared to be an appropriate candidate for thrombolysis based on the timing of symptom onset and a negative noncontrast head CT. However, thrombolytic therapy, including TNK, is contraindicated in patients with active internal bleeding [11,12]. The discovery of an AAA with signs of rupture on point-of-care ultrasound and confirmatory imaging appropriately precluded thrombolytic therapy. This case therefore emphasizes that, while rapid treatment is critical in stroke care, clinicians must remain vigilant for contraindications and alternative diagnoses before administering thrombolytics. Specifically, if there is high suspicion for internal bleeding or another contraindication to thrombolytic therapy, imaging studies should be performed to rapidly evaluate for these conditions before administration of thrombolytics.

Management following diagnosis also posed challenges due to conflicting hemodynamic goals between stroke care and postoperative AAA repair. In patients with acute ischemic stroke who have not received thrombolytic or other reperfusion therapy, permissive hypertension is recommended to maintain cerebral perfusion, with systolic blood pressures tolerated up to 220 mmHg [11]. In contrast, after repair of a ruptured AAA, particularly following EVAR, blood pressure is typically controlled lower and more tightly to reduce stress on the vascular repair and minimize the risk of bleeding or graft complications [13]. In our patient, postoperative systolic blood pressure targets of 100-130 mmHg were maintained following endovascular repair. While these goals may be lower than those typically tolerated in acute stroke management, they were necessary to protect the repaired aneurysm. As a result, optimal care required multidisciplinary coordination between the vascular surgery and neurology teams to balance the competing risks of hemorrhage and cerebral hypoperfusion. 

## Conclusions

This case highlights the competing aims of obtaining a rapid neurologic evaluation and proceeding expeditiously toward time-sensitive therapies such as thrombolytics while simultaneously screening for contraindications to their use. Clinicians should maintain a broad differential diagnosis when evaluating patients with acute neurologic deficits, particularly when symptoms such as chest, back, or abdominal pain precede or accompany neurologic findings. Moreover, concurrent management of stroke and major aortic pathology may require individualized hemodynamic strategies, as standard treatment recommendations for each condition may conflict. Early recognition and multidisciplinary collaboration are therefore essential to optimizing outcomes in these complex clinical scenarios.
